# Genome-Wide Identification of GDSL-Type Esterase/Lipase Gene Family in *Dasypyrum villosum* L. Reveals That *DvGELP53* Is Related to BSMV Infection

**DOI:** 10.3390/ijms222212317

**Published:** 2021-11-15

**Authors:** Heng Zhang, Xu Zhang, Jia Zhao, Li Sun, Haiyan Wang, Ying Zhu, Jin Xiao, Xiue Wang

**Affiliations:** 1 State Key Laboratory for Managing Biotic and Chemical Threats to The Quality and Safety of Agro-Products, Institute of Virology and Biotechnology, Zhejiang Academy of Agricultural Sciences, Hangzhou 310021, China; zhangheng20170927@163.com (H.Z.); yzhuzaas@163.com (Y.Z.); 2 State Key Lab of Crop Genetics and Germplasm Enhancement, Cytogenetics Institute, Nanjing Agricultural University/JCIC-MCP, Nanjing 210095, China; 2018201061@njau.edu.cn (X.Z.); sunli@njau.edu.cn (L.S.); hywang@njau.edu.cn (H.W.); xiaojin@njau.edu.cn (J.X.); 3 College of Agriculture, South China Agriculture University, Guangzhou 510642, China; zhaojia@scau.edu.cn

**Keywords:** GELP, gene family, *Dasypyrum villosum* L., virus movement

## Abstract

GDSL-type esterase/lipase proteins (GELPs) characterized by a conserved GDSL motif at their N-terminus belong to the lipid hydrolysis enzyme superfamily. In plants, GELPs play an important role in plant growth, development and stress response. The studies of the identification and characterization of the *GELP* gene family in Triticeae have not been reported. In this study, 193 *DvGELPs* were identified in *Dasypyrum villosum* and classified into 11 groups (clade A–K) by means of phylogenetic analysis. Most *DvGELPs* contain only one GDSL domain, only four *DvGELPs* contain other domains besides the GDSL domain. Gene structure analysis indicated 35.2% *DvGELP* genes have four introns and five exons. In the promoter regions of the identified *DvGELPs*, we detected 4502 putative *cis*-elements, which were associated with plant hormones, plant growth, environmental stress and light responsiveness. Expression profiling revealed 36, 44 and 17 *DvGELPs* were highly expressed in the spike, the root and the grain, respectively. Further investigation of a root-specific expressing *GELP*, *DvGELP53*, indicated it was induced by a variety of biotic and abiotic stresses. The knockdown of *DvGELP53* inhibited long-distance movement of BSMV in the tissue of *D. villosum*. This research provides a genome-wide glimpse of the *D. villosum GELP* genes and hints at the participation of *DvGELP53* in the interaction between virus and plants.

## 1. Introduction

The GDSL-type esterase/lipase protein (GELP, esterase, EC 3.1.1) is a newly discovered lipid hydrolysis enzyme (lipolytic enzyme). They are featured in having a conserved GDSL motif at the N-terminus, whereas many other lipases have the GxSxG motif near their protein center [1,2]. GELPs have a consensus sequence that is divided into five conserved sequence blocks (I–V) and four invariant important catalytic residues serine (Ser), glycine (Gly), asparagine (Asn) and histidine (His) in blocks I, II, III and V, respectively [1]. The active-site Ser in block I and the Asp, His residues in block V compose a catalytic triad existing in all GELPs. The Gly residue in block II and the Asn in block III act as proton donors to the oxyanion hole [1,2]. GDSL lipases were widely found in prokaryotes and eukaryotes [3]. To date, GELP family genes have been well-studied at the genome-wide level in various species. A total of 105, 114,194, 114, 96, 126 and 130 *GELP* genes have been identified in *Arabidopsis thaliana*, *Oryza sativa*, soybean, *Brassica rapa*, *Vitis vinifera*, *Populus trichocarpa* and *Sorghum bicolor*, respectively [3,4,5,6].

The functions of *GELPs* have been extensively studied in *Arabidopsis* [7], *Oryza* [8], pepper [9] and other plants. They are mainly involved in the regulation of plant development, morphogenesis, synthesis of secondary metabolites and defense response. *AtEXL4* from *Arabidopsis* [10], *ZmMS30* from maize [11], *OsGELP34*, *OsGELP110* and *OsGELP115* from rice [12] were proven to regulate the pollen development. *Arabidopsis* GDSL-type esterase gene *AtSFAR4* (seed fatty acid reducers) is intensely expressed in embryo protrusion, early seedlings and pollen and plays a role in enhancing the seed germination rate through upregulating fatty acid degradation-related genes [13]. The *BS1* from *Oryza* encoding a GDSL lipase plays an important role in the maintenance of proper acetylation level on the xylan backbone, which is crucial for secondary wall formation and patterning [14]. As a sinapine esterase involved in secondary metabolism, GDSL lipase-like genes, such as *BnSCE3*/*BnLIP2* in Brassicaceae, catalyze hydrolysis of sinapine during seed germination [15]. More and more evidence is accumulated for the critical role of *GELP* in biotic stress response through hormone signaling regulation. In *Arabidopsis*, *GLIP1* modulates resistance of multiple pathogens by manipulating ethylene (ET) signaling [16,17]. The pepper *CaGLIP1* expression was induced by salicylic acid (SA), ET, methyl jasmonate (MeJA) and abiotic stress. Knockdown of *CaGLIP1* in pepper conferred enhanced resistance to *Xcv (Xanthomonas campestris* pv. *vesicatoria)*, while overexpression of *CaGLIP1* in *Arabidopsis* compromised the resistance to the bacterial pathogen *Pseudomonas syringae* pv. *tomato* [9]. In rice, *OsGLIP1* and *OsGLIP2* were suppressed by pathogen infection and SA treatment. Increased expression of *OsGLIP1* and *OsGLIP2* compromised the disease resistance in rice, while knocking them down elevated the resistance to bacterial and fungal pathogens [8]. Moreover, *OsGLIP2* contributed resistance to *Erwinia carotovora* via negatively regulating auxin signaling [18]. Besides, recent studies have shown that *GELPs* are also involved in abiotic stress response in plants. For example, overexpression of *AtSFAR4* enhanced the osmosis tolerance to glucose [13]. Transgenic *Arabidopsis* overexpressing *AtLTL1,* which was rapidly induced by LiCl and NaCl, elevated the salt tolerance [7]. The *CaGLIP1* transgenic pepper showed drought tolerance during seed germination and the plant growth stage [9]. All these results indicated that GELPs are important enzymes in plants and may have multiple functions in regulating various physiological processes.

Viruses can cause diseases in a variety of crops and reduce their yield and quality, such as in wheat, barley and rice [19]. After invasion, viruses rapidly spread to the whole plant and cause disease symptoms. The virus movement in plants is generally divided into two aspects: cell-to-cell through plasmodesmata and long-distance through the phloem [20]. Genes related to virus movement have been identified both in plants and viruses. In viruses, the 37 kD MP (movement proteins) from SBWMV (*Soil*-*borne wheat mosaic virus*) and CI (cylindrical inclusion) from WYMV (*Wheat yellow mosaic virus*) were shown to mediate the cell-to-cell and long-distance virus movement [21,22,23]. P3N-PIPO and VPg (viral genome-linked protein) from several potyvirus species have been proven to participate in virus movement as well [24,25,26]. However, only a few plant proteins have been identified to be involved in virus movement. For example, NbHIPP26 from *Nicotiana benthamiana* (*N. benthamiana*) interacted with the movement protein TGB1 from potato mop-top virus to facilitate long-distance virus movement [27]. The interaction between VPg and PVIP (*Potyvirus* VPg-interaction protein) may modulate expression of host genes involved in virus movement [28,29]. To better understand the plant–virus interaction and the molecular mechanism of plant defense against the virus, more genes related to virus movement should be identified in plants.

Common wheat (*Triticum aestivum* L. AABBDD, 2*n* = 6*x* = 42) is a hexaploid with a huge and complex genome [30,31,32]. This causes difficulties in the analysis of gene families and functions. *Dasypyrum villosum* L. (*D. villosum*, 2*n* = 2*x* = 14, VV), also known as *Haynaldia villosa*, is a diploid wild relative of wheat [33]. *D. villosum* possesses high resistance or tolerance to abiotic and biotic stresses, such as powdery mildew [34,35], yellow rust [36], WYMV [37] salt [38] and so on. It is an excellent genetic resource for wheat improvement. The purpose of this study was as follows: understand the evolution and function of the *GELP* family genes in *D. villosum* and discover the genes regulating virus resistance. We performed genome-wide identification of *GELP* in *D. villosum*. The evolutionary relationship, chromosome distribution and protein structure of GELPs were further analyzed in *D. villosum*. *DvGELP53*, a *GELP* specifically expressed in the root, was chosen for further characterization to elucidate its function in response to virus infection using a barley stripe mosaic virus (BSMV)-induced gene silencing system. Our results will facilitate the understanding of the evolution, diversification and function of *GELPs* in *D. villosum*.

## 2. Results

### 2.1. Genome-Wide Identification of the GELP Gene Family in D. villosum

**Figure 1:**
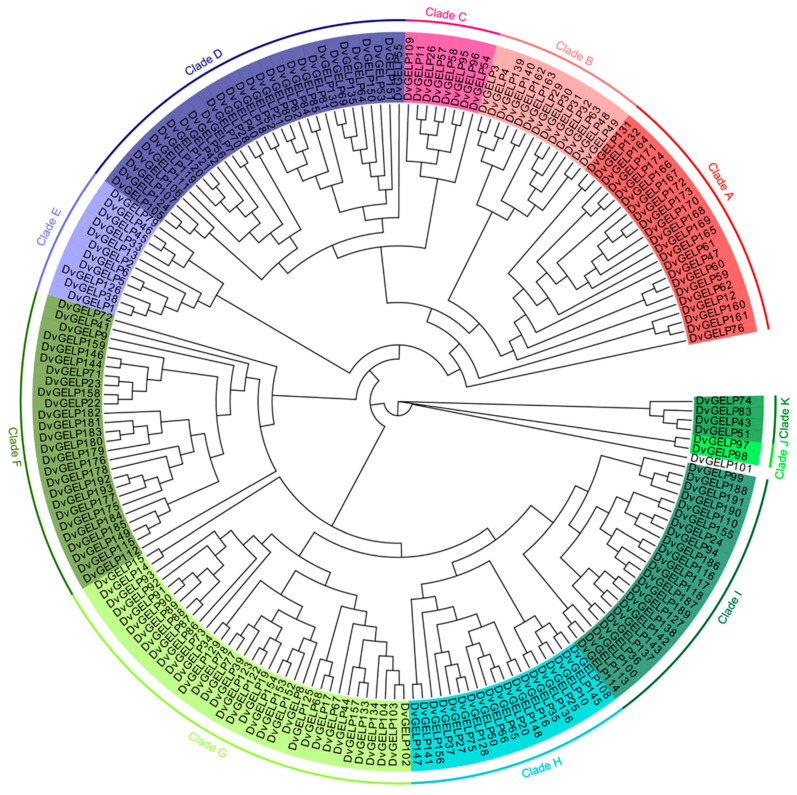
The phylogenetic tree of the *GELP* gene family from *D. villosum*.

A total of 193 *GELP* family genes were identified in the genome of *D. villosum*; they were designated *DvGELP1*–*DvGELP193* according to their chromosomal locations (Table S1)*. DvGELP83* has the shortest CDS of 717 bp and encodes a protein of 238 amino acids (aa), while *DvGELP60* has the longest CDS of 2742 bp and encodes a 913 aa protein. A phylogenetical neighbor-joining tree of *DvGELPs* was built; *DvGELPs* could be divided into 11 clades. Clades A–K had 22, 13, 8, 30, 11, 26, 34, 19, 23, 2, 4 *DvGELPs*, respectively. Clade J was the smallest clade and had only two members while Clade G was the largest and had 34 members (Figure 1). The numbers of *DvGELPs* were quite different across clades, implying the functional divergence of these genes.

DvGELPs were analyzed for their conserved domains using the Conserved Domain Database. All the predicted DvGELPs had one lipase GDSL domain, and only four DvGELPs had other functional domains. DvGELP36 and DvGELP188 had an SGNH_hydrolase superfamily domain, DvGELP37 had an ATP-synt_Z domain, and DvGELP52 had a Glyco_hydro superfamily and X8 domain (Figure S1).

The exon–intron structure of the *DvGELPs* belonging to different subgroups was analyzed and compared (Figure S1). Three *DvGELPs* (*DvGELP19*, *DvGELP50* and *DvGELP83*) had no introns, and the number of introns of the remaining genes ranged from one to 14. *DvGELP*s with four introns are the most common in this gene family (68 out of 193, 35.2%), followed by those having three and two introns, which account for 15.0% (29 out of 193) and 20.2% (39 out of 193), respectively. *DvGELPs* containing eight, ten and 14 introns account for 1.0%, 1.0% and 0.5%, respectively (Table S1). These results are consistent with the previous rule that *GELPs* with four introns are the majority in plants. For example, the members of *GELP* with four introns were approximately 67.6%, 49.1% and 74.2% in *Arabidopsis*, rice and soybean, respectively. The number of introns of *DvGELPs* within a closer phylogenetic relationship is generally similar, but exceptions were also observed for some genes. For instance, *DvGELP21* and *DvGELP80* belong to the same branch of Class D; the former has seven introns while the latter has two introns (Figure S1). The difference in gene structure may reveal the evolutionary and functional differentiation of *DvGELP* genes across different subgroups as well as within the same subgroup.

### 2.2. Chromosomal Location of DvGELPs

**Figure 2:**
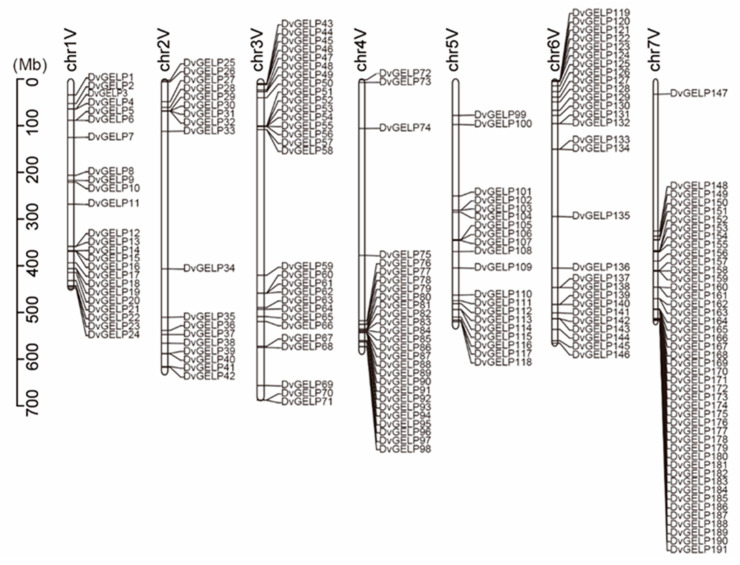
Chromosomal location of *DvGELPs* in *D. villosum*. The identities of the chromosome are indicated at the top of each chromosome, while the *GELP* gene names are shown to the right of each chromosome.

The chromosomal location analysis revealed the *DvGELPs* were distributed on all the seven chromosomes and two scaffolds of the *D. villosum* genome (*D. villosum* sequence was accomplished but not released). The number of *DvGELP* genes on different chromosomes varied. There were 24, 18, 29, 27, 20, 28 and 45 *DvGELPs* in the chromosomes 1–7V, respectively. Chromosome 7V had the most (23.3%) and 2V had the least *DvGELPs* (9.3%). *DvGELPs* were mainly located in the distal region of chromosome arms, and only a few genes were in the middle of the chromosomes 1V and 5V (Figure 2). This result is similar to that in soybeans in that 78% of the predicted genes are located at the distal end of each chromosomal end. We also found that *DvGELPs* were mostly present as clusters, such as *DvGELP12-24* in chromosome 1V, *DvGELP76-98* in 4V and *DvGELP148-191* in 7V. We speculate that this phenomenon may be caused by gene duplication during evolution.

### 2.3. Promoter Sequence Analysis and Expression Profiles of DvGELPs

**Figure 3:**
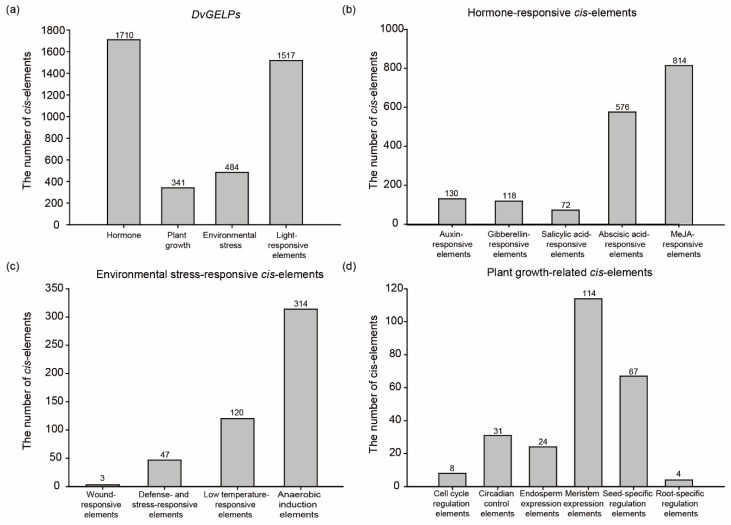
The *cis*-elements analysis of the *DvGELPs* promoter sequence. (**a**) The total number of the four types of elements. (**b**) The classification and number of hormone-responsive elements. (**c**) The classification and number of environmental stress-responsive elements. (**d**) The classification and number of plant growth-related elements.

The *cis*-elements in the promoter region play important roles in regulating gene transcription when challenged by biotic and abiotic stresses [6]. The 2.0 kb upstream sequence from the translational start site of *DvGELPs* was downloaded and analyzed using PlantCARE. A total of 4502 *cis*-elements were detected in the promoter region of the 193 *DvGELP* genes. These *cis*-elements included plant hormones, plant growth, environmental stress and light-responsive elements (Figure 3a). The hormone-related *cis*-elements were the most common (1710, 38.0%), in which 130 were auxin (IAA)-responsive, 118 were gibberellin (GA)-responsive, 72 were SA-responsive, 576 were abscisic acid (ABA)-responsive and 814 were MeJA-responsive elements (Figure 3b), followed by 1517 (33.7%) light-responsive elements (Figure 3a). In the 484 (10.8%) environmental stress elements, three were wound-responsive, 47 were defense- and stress-responsive, 120 were low temperature-responsive and 314 were anaerobic induction elements (Figure 3c). Plant growth-related *cis*-elements had the least number of 341 (7.6%), in which eight were cell cycle regulation, 31 were circadian control, 24 were endosperm expression, 114 were meristem expression, 67 were seed-specific regulation and four were root-specific regulation elements (Figure 3d). These results reveal that the promoter of *DvGELP* genes may be involved in plant growth, development and interaction with various environmental stresses, further suggesting the functional diversity of the *DvGELP* gene family.

**Figure 4:**
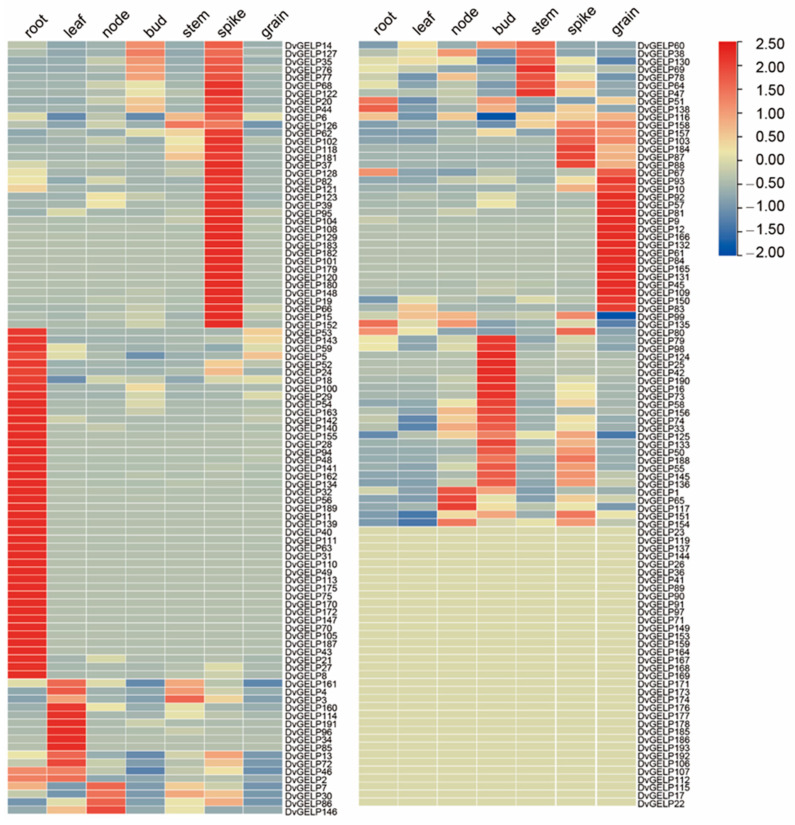
Heatmap of the expression profiling of *D. villosum GELPs* in different tissues. The expression signal of each gene was based on the Z-score normalization value from the RNA-seq data. The root, leaf, node, bud, stem and spike were sampled at 20 d after flowering, and the grain was sampled at the maturity stage of *D. villosum*. Each sample has three biological replicates. RNA-Seq was performed using a BGISEQ-500 sequencing platform by BGI Company, Shenzhen, China.

The expression pattern of different gene members may provide clues of their conserved or diversified biological function. To preliminarily predict the function of *DvGELP*s, we investigated their expression in different *D. villosum* tissues by RNA-seq. The results showed that 36 *DvGELP*s were highly transcripted in the spike (e.g., *DvGELP152*), 44—in the root (e.g., *DvGELP27*), six—in the leaf (e.g., *DvGELP85*), 17—in the grain (e.g., *DvGELP83*), seven—in the stem (e.g., *DvGELP47*), 12—in the tiller bud (e.g., *DvGELP33*) and seven—in the node (e.g., *DvGELP7*) (Figure 4). A small portion of genes was highly expressed in two tissues. *DvGELP138* was highly expressed both in the root and the bud, and *DvGELP72* was highly expressed both in the spike and the leaf (Figure 4). A few genes were constitutionally expressed in *D. villosum*. For instance, *DvGELP99* and *DvGELP*135 exhibited high transcription levels in most tissues (Figure 4). The expression of 35 genes (18.1%) could not be detected in any tissue (i.e., *DvGELP22*) (Figure 4), indicating that they may not be expressed in the examined tissue or their expression may be inducible by environmental factors.

### 2.4. DvGELP53 Plays Role in Long-Distance BSMV Movement in D. villosum

From the above results, we found that the promoter sequence of *DvGELP53* included plant hormones and plant growth elements, such as MeJA-responsive, ABA-responsive and meristem expression-related elements. The expression profiling of *DvGELPs* showed *DvGELP53* is specifically and highly expressed in roots. Previous studies reported that root-specific expression genes may be involved in plant–virus interaction [27]. We further investigated the role of *DvGELP53* in response to virus infection.

**Figure 5:**
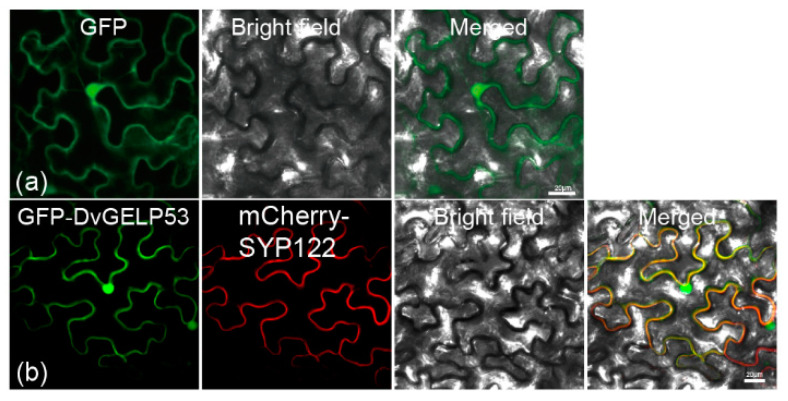
Subcellular localization of DvGELP53 in the epidermal cells of *N. benthamiana*. (**a**) GFP and (**b**) GFP-DvGELP53; GFP was used as the control. The localization of mCherry-SYP122 is shown in red, and the localization of GFP and its fusion proteins is shown in green. Scale bar = 20 µm.

*DvGELP53* belongs to Clade D encoding for 1212 bp nucleotide and 403 amino acids which contains an SGNH/GDSL hydrolase domain (A^38^ to P^369^). The protein has a predicted molecular weight of 44.14 kD and isoelectric point of 8.16. DVGELP53 was subcellularly localized to the plasma membrane and in the nucleus in the epidermal cells of *N. benthamiana* (Figure 5b), confirmed by the signal overlapping of GFP-DvGELP53 and plasma membrane marker protein mCherry-SYP122 (Figure 5b).

**Figure 6:**
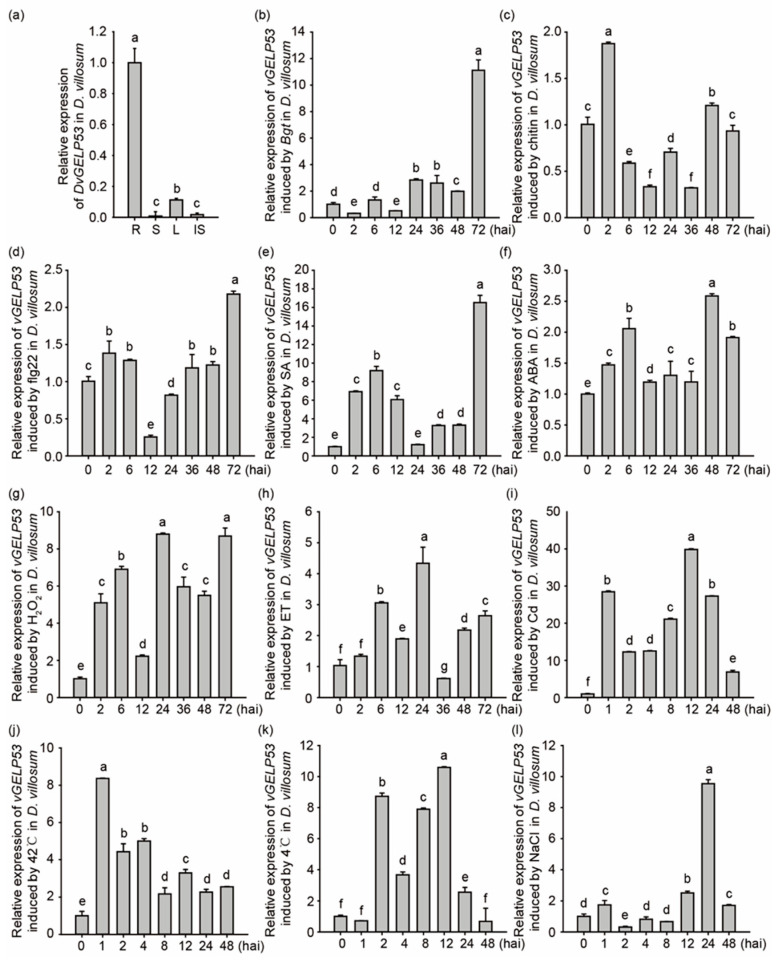
Expression profiling of *DvGELP53*. (**a**) The expression of *DvGELP53* in the root (R), the stem (S), the leaf (L) and the immature spike (IS) of *D. villosum*. The expression in the root is set to 1. Time course expression profiling of *DvGELP53* in the leaf of *D. villosum* in response to *Bgt* (**b**), chitin (**c**), flg22 (**d**), SA (**e**), ABA (**f**), H_2_O_2_ (**g**), ET (**h**), Cd (**i**), 42 °C (**j**), 4 °C (**k**) and NaCl (**l**). The expression at 0 h is set as 1. Bars indicate the standard error (SE), bars with different letters show significant differences at *p* < 0.05.

The expression profiling of *DvGELP53* was further validated by qRT-PCR. The tissue-specific expression pattern of *DvGELP53* in *D. villosum* was confirmed, shown by that it had the highest transcription level in the root, followed by that in the leaf and a very low level in the stem (Figure 6a). In the *D. villosum* leaf, *DVGELP53* was downregulated at 2 and 12 h after inoculation (hai) of *Blumeria graminearum* f. sp. *tritici* (*Bgt*), upregulated from 24 hai and reached a peak at 72 hai (Figure 6b). *DVGELP53* was quickly accumulated when treated by means of two pathogen-associated molecular patterns (PAMP), fungal chitin and bacterial flg22 treatment. Its expression reached a peak at 2 and 72 h after treatment, respectively (Figure 6c,d). These indicated that *DvGELP53* may be involved in the response of wheat to biotic stresses. *DVGELP53* expression in the *D. villosum* leaf was also induced by exogenous application of phytohormones such as SA (5 mM), ABA (0.2 mM), ET (0.1 mM) and H_2_O_2_ (7 mM) as well as abiotic stresses such as heavy metal cadmium (Cd, 1 mM), heat (42 °C), cold (4 °C) and salt (NaCl, 100 mM) (Figure 6e–l). These indicated that *DvGELP53* participates in the regulation of phytohormone pathways and abiotic stress responses.

**Figure 7:**
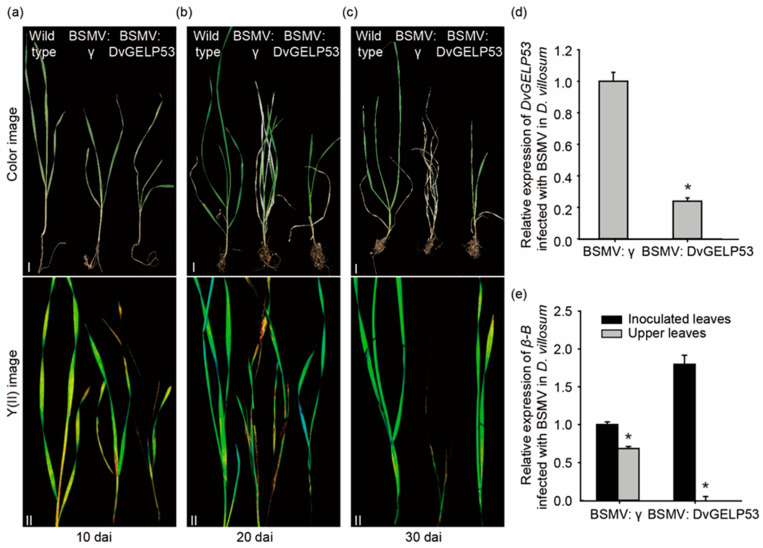
Functional analysis of *DvGELP53* by virus-induced gene silencing (VIGS). *D. villosum* leaves of the seedlings inoculated with BSMV:γ or BSMV:DvGELP53 at 10 days after inoculation (dai) (**a**), 20 dai (**b**) and 30 dai (**c**). Successful virus infection in *D. villosum* was verified by detecting the chlorotic mosaic phenotype on the leaves; (I) and (II) represent visual observation and false-color images of Y(II) measurements at different inoculation times, respectively. Wild type and BSMV:γ were control. (**d**) Expression levels of *DvGELP53* in the BSMV:γ and BSMV:DvGELP53 plants. The BSMV:γ is set to 1. (**e**) Expression levels of *β-B* (from the BSMV genome) in inoculated leaves and upper leaves of *D. villosum* at 20 dai. Each point represents the mean of three replicates. Bars indicate the standard error (SE), and * show significant differences at *p* < 0.05.

To examine the role of *DvGELP53* in plant–virus interaction, we used a BSMV-mediated virus-induced gene silencing (VIGS) system to knock down the expression of *DvGELP53* in *D. villosum* (Figure 7(aI)); qRT-PCR confirmed the expression of the target gene *DvGELP53* was significantly downregulated in the third leaves of BSMV:DvGELP53-inoculated plants compared with that in the BSMV:γ control (Figure 7d). This indicated *DvGELP53* was silenced successfully. To validate the presence of BSMV, the *β-B* of BSMV in the inoculated leaf (the third leaf) and the upper systemic leaf (the fourth leaf) were detected. We detected *β-B* in the third leaves from both the BSMV:γ- and BSMV:DvGELP53-inoculated plants. However, we detected *β-B* only in the upper systemic leaves (the fourth leaves) of the plants inoculated with BSMV:γ but not with BSMV:DvGELP53 (Figure 7e). We suspect the knockdown of *DvGELP53* blocked the movement of BSMV from the third leaf (inoculated leaf) to the fourth leaf (systemic leaf). We further compared the phenotype brought by BSMV infection on leaves of the plants inoculated with BSMV:γ (control) and BSMV:DVGELP53. At 10 dai of BSMV, the chlorotic symptom was observed on the third leaves (inoculated leaf) of the plants inoculated with either BSMV:γ or BSMV:DvGELP53, with the symptom more pronounced in case of BSMV:DVGELP53 (Figure 7(aI)). At 20 dai, obvious chlorosis was observed on more upper leaves (the non-inoculated fourth and fifth leaves) in the BSMV:γ control, while chlorosis was restricted and only observed in the inoculated leaf (the third leaf) in case of BSMV:DVGELP53 (Figure 7(bI)). At 30 dai, the BSMV:γ plant was completely BSMV-infected and lethal while the BSMV:DvGELP53 plant had no obvious chlorosis (Figure 7(cI)). False-color images of Y (II) further confirmed the above difference between BSMV:γ (control) and BSMV:DVGELP53 (Figure 7(aII–cII)). These results indicated that knockdown of *DvGELP53* inhibited the movement of BSMV from inoculated leaves to non-inoculated upper leaves in *D. villosum*.

### 2.5. Preliminary Study of the Mechanism of DvGELP53 in Regulating Long-Distance BSMV Movement in D. villosum

**Figure 8:**
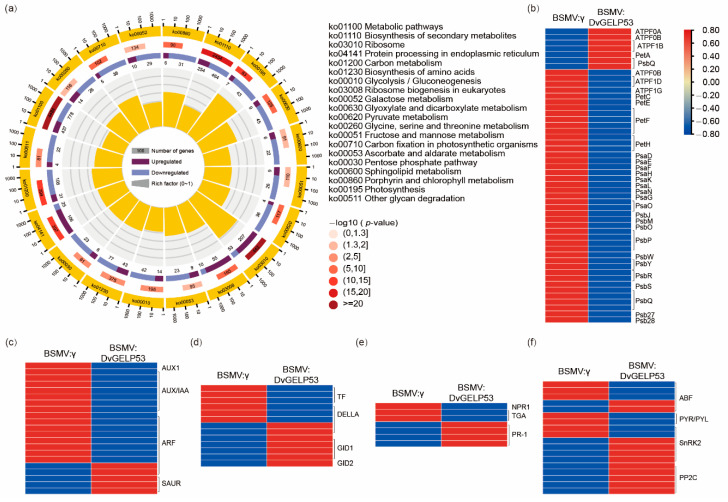
Transcriptome analysis of BSMV:γ and BSMV:DvGELP53. (**a**) KEGG pathway analysis of BSMV:γ and BSMV:DvGELP53 for the third leaves inoculated with virus. The circular chart represents distinct KEGG pathways of differentially expressed genes (DEGs). The graph is divided into four circles from the outside to the inside. The first circle represents the terms of enrichment and the outside of the circle is the coordinate ruler of the number of genes. The second circle represents the number of background genes in the category and the *p*-value. The length of the bar represents the number of genes and the shade of red represents the size of the *p*-value. The third circle represents the ratio of the number of upregulated and downregulated genes, dark purple and light purple represent upregulated and downregulated genes, respectively, and the number of genes is shown below. The fourth circle represents the rich factor value of each category, and each box in the background represents 0.1. Heatmap of photosynthesis (**b**), IAA (**c**), GA (**d**), SA (**e**) and ABA (**f**) pathway DEGs in case of BSMV:γ and BSMV:DvGELP53. The expression signal of each gene is based on the Z-score normalization value.

To further investigate the function of *DvGELP53* in the regulation of virus movement in plants, genome-wide gene expression patterns were compared between BSMV:γ and BSMV:DvGELP53 using the third leaves at seven dai of virus. At seven dai, the two types of plants had obvious phenotypic difference, while the third leaves of the BSMV:DvGELP53 plants had not withered yet. A total of 7655 differentially expressed genes (DEGs) with at least a twofold change (*p* < 0.001) were identified in case of BSMV:DvGELP53, in which 3942 were upregulated and 3713 were downregulated genes. The Kyoto Encyclopedia of Genes and Genomes (KEGG) analysis showed that the DEGs were significantly enriched in terms of plant growth and development, such as “metabolic pathways”, “biosynthesis of secondary metabolites”, “carbon metabolism”, “carbon fixation in photosynthetic organisms”, “porphyrin and chlorophyll metabolism” and “photosynthesis” (Figure 8a). Only three enriched terms had more upregulated than downregulated genes (i.e., ko03008). The remaining 17 terms had more downregulated than upregulated genes. Among the 40 DEGs in the photosynthesis pathway (ko00195), seven genes were upregulated and 43 were downregulated, and the expression changes are shown in the heatmap (Figure 8a,b).

The Gene Ontology (GO) analysis showed that the DEGs were also significantly enriched in plant growth and development, such as “translation”, “organonitrogen compound biosynthetic process”, “photosynthesis”, “electron transport chain”, “nitrogen cycle metabolic process” and “homeostatic process” (Figure S2). The plant hormone (IAA, GA, SA and ABA) signaling pathway genes were analyzed in the transcriptome data. For the IAA signaling pathway, there were more downregulated genes than upregulated ones. In *DvGELP53*-silenced *D. villosum, AUX1*, *AUX/IAA* and *ARF* were downregulated and ARF, SAUR were upregulated (Figure 8c). For the GA, SA and ABA signaling pathways, there were more upregulated genes identified than downregulated genes. *PR-1* and *PP2C* were both upregulated in *DvGELP53*-silenced *D. villosum* (Figure 8d–f). These results showed a large amount of genes related to plant growth and development were rapidly downregulated, and the expression levels of the plant hormone signaling pathways were also changed in *DvGELP53*-silenced *D. villosum*, causing chlorosis and wilting of the virus-inoculated leaves, and inhibited the virus movement to other leaves, resulting in the prevention of the virus spreading throughout the plants.

## 3. Discussion

### 3.1. DvGELP in D. villosum May Have Been Duplicated during Evolution

GDSL esterase/lipase proteins (GELP) are a subfamily of lipolytic enzymes and the *GELP* gene family has been identified in only a few plants, such as model plant *Arabidopsi*s, rice and oil crop soybean. Wheat is an important food crop around the world and no *GELP* gene family studies have been found on wheat and its related species. In this study, we identified and characterized the *GELP* gene family in *D. villosum*. *D. villosum* is a diploid plant similar to *Arabidopsi*s and rice, and 105 and 114 *GELPs* were found in *Arabidopsi*s and rice, respectively. However, *D. villosum* with 193 GELPs is similar to soybean (ancient paleopolyploid) with 194 GELPs [6]. Su et al. found that the *GELP* genes of soybean are duplicated by collinearity alignment and nonsynonymous/synonymous substitution ratio (Ka/Ks) analysis. In rice, approximately 47% of the *OsGELP* genes are closely arranged on chromosomes, comprising 17 clusters [3]. In the soybean genome, 71% of the *GmGDSL* family members occur as WGD (whole-genome duplication)/segmental duplication. In our research, the chromosome location analysis showed *DvGELPs* were mainly closely distributed at the ends of chromosomes, and we speculated about *DvGELP* duplication during evolution. On average, 65% of genes have a duplicate copy in plant genomes, and these duplications may lead to the generation of novel functions, such as the adaptability of plants to environmental stress [39,40]. For example, *Arabidopsis* duplicates have likely accumulated novel environmental responses and novel developmental regulatory patterns [41,42]. The duplication of disease resistance (R) genes and prolamin genes in plants is also a common phenomenon, which is closely related to their function [43,44]. Analysis of the *GELP* gene family in different plants shows that the GELP also features duplication, and this duplication may be related to the biological function of the GELP [3,6].

### 3.2. The GELP Gene Family May Regulate Plant Development and Various Stress Responses in D. villosum

The roles of the GELP in plant growth and development, organ morphogenesis, secondary metabolism and stress resistance have been demonstrated. The *GELP* gene family analysis of different species shows that the expression of *GELP* is very different. In *Arabidopsis*, six *AtGELPs* are expressed in the reproductive organs (siliques and seeds), five *AtGELPs* appear specifically expressed in the roots, and 16 genes are exclusively expressed in flowers [4]. Some research showed that the function of the *GELP* gene in *Arabidopsis* is closely related to its specific expression, such as that many *GELP* genes have been confirmed to be involved in the development of pollen, including *EXL4*, *EXL6* and *GELP77* [45] in *Arabidopsis* and *OsGELP34* [10,46]. *EXL4* is specially expressed in anthers of development flowers [47], which affect lipid composition of the pollen coat, and loss of *EXL4* leads to the delay in pollen hydration, which further reduces the competitiveness of pollen when compared with wild-type pollen [10]. *EXL6* was upregulated during the early stage of pollen development and knockdown of *EXL6* caused pollen cytoplasmic degradation after the tetrad stage [46,48]. In rice, most of the *OsGELP* genes are expressed in various organs (i.e., *OsGELP1*), and some genes are only expressed in a specific tissue. i.e., *OsGELP68* is only expressed in panicles. *OsGLIP1* is mainly expressed in the leaf and leaf sheath, and *OsGLIP2* is highly expressed in elongating internodes. In soybean, a small portion of *GmGELP* are expressed constitutionally (e.g., *GmGELP22*), and large amounts of genes show tissue-specific expression, e.g., *GmGELP10* shows preferential expression in young leaves, flowers, pods and pod shells. In our study, *GELPs* from *D. villosum* showed different expression patterns, for example, 36 and 44 *DvGELP*s exhibited high transcription levels in the spike and the root, respectively, and *DvGELP138* was highly expressed in the root and the bud. Specific tissue expression is directly related to their function.

The expression of *GELP* is also induced by various hormones and stress. The *OsGLIP1* and *OsGLIP2* from rice are suppressed by pathogen infection and salicylic acid (SA) treatment and negatively regulate plant resistance to bacterial and fungal pathogens. The GER1 from rice is rapidly induced by early light and jasmonic acid (JA) as a negative regulator of light-triggered biosynthesis and coleoptile elongation [49]. The *CaGLIP1* from pepper is induced by SA, ET, MeJA, sodium nitroprusside, methyl viologen, high salt, mannitol-mediated dehydration and wounding [9]. Overexpression of *CaGLIP1* can enhance the tolerance of transgenic *Arabidopsis* to drought and susceptibility to the bacterial pathogen *Pseudomonas syringae* pv. *tomato* [9]. In our research, the *cis*-element of the *DvGELP* promoter sequence included plant hormones, plant growth, environmental stress and light-responsive elements, and the ratio of each component was different. This reveals that the expression of *DvGELP* can be induced by various biological processes and stress. *DvGELP53* was mainly expressed in the root, and was induced by many biotic and abiotic stresses and plant hormones (i.e., powdery mildew, NaCl and SA); silencing *DvGELP53* in *D. villosum* may inhibit the movement of BSMV. This result shows that *DvGELP* is involved in the interaction of plants and viruses. It implies that the *DELP* genes might be involved in plant development processes as well as multiple stress responses in *D. villosum*.

### 3.3. DvGELP53 May Function in Regulating Virus Movement and Can Be a Gene Resource for Virus Disease Resistance

After the virus infects the plant, the systemic infection of plants develops through virus’s cell-to-cell movement and long-distance movement [20]. At present, genes related to virus movement have been found in viruses and plants. The movement proteins (MP) from SBWMV, CI from WYMV, P3N-PIPO from TuMV (*Turnip mosaic virus*) were all proved to participate in the movement of viruses in plants. Previous studies showed that viruses mainly move through plasmodesmata (PD) in plants; the PD is essential for systemic infection in plants by viruses. CI mutations that disrupt virus cell-to-cell movement compromise PD localization capacity [24]. Few genes are involved in virus movement found in plants, and they are mainly found in model plants, e.g., *Arabidopsis* and *N. benthamiana*. NbHIPP26 from *N. benthamiana* is mainly expressed in root tissues and interact with TGB1 (from PMTV) to participate in the long-distance movement of the PMTV. In *NbHIPP26*-silenced plants, PMTV cannot move to the leaves that are not inoculated with the virus [27]. NbRPL1 (ribosomal protein large subunit 1) interacts with NIb (mosaic virus (TVBMV) nuclear inclusion protein) in the chloroplasts, enhancing TVBMV infection. After inoculating TVBMV-GFP in *NbRPL*1-silenced *N. benthamiana* plants, the green fluorescence and TVBMV CP cannot be detected in the systemic leaves of the NbRPL1-silenced plants, only in control plants [50]. The auxin/indole acetic acid (Aux/IAA) transcriptional regulators IAA18, IAA26 and IAA27 interact with the tobacco mosaic virus (TMV) 126/183-kDa replicase [51]; a study showed that TMV infection inhibits the nuclear localization of the Aux/IAA proteins, which correlates with enhanced TMV movement and spread in the phloem of older leaves [52]. Wheat is the second most important cereal crop in the world, and soil-borne bymoviruses cause yield losses in wheat as a group of agronomically important pathogens [53]. The study showed that the TaLIP (light-induced protein) interacts with the WYMV nuclear inclusion b protein (NIb); the expression of *TaLIP* was decreased by WYMV infection, and knockdown of *TaLIP* compromised wheat resistance to WYMV [54]. In our research, *DvGELP53* was cloned from the *D. villosum* relative of wheat, which is highly expressed in roots, similarly to *NbHIPP26*. Subcellular location shows that DvGELP53 is mainly located in the nucleus and the plasma membrane. The nucleus is shown to play a role in the long-distance movement of some plant viruses [55]. In *DvDELP53*- silenced plants, the virus RNA (β-B) cannot be detected in uninoculated leaves, indicating that the long-distance movement of the virus is inhibited.

The chloroplasts play an important role in plant–virus interaction, and chloroplast proteins were proved to be involved in several viral infection processes [50]. Chloroplast proteins such as glyceraldehyde 3-phosphate dehydrogenase subunit A (NbGAPDH-A) [56] and chloroplast phosphoglycerate kinase (chl-PGK) [57] are both proven to be involved in virus replication and movement. In our study, transcriptome analysis results showed that many DEGs associated with photosynthesis were mostly downregulated in *DvGELP53*-silenced plants, and most of these genes play a role in chloroplasts. Therefore, we speculate that *DvDELP53* may affect the long-distance movement of BSMV by regulating the expression of certain genes in the chloroplasts. SA and ABA may be related to the movement of plant viruses as exemplified in several studies [28]. In *N. tabacum* plants, the *plum pox virus* (PPV) replicates and moves from cell to cell, but does not spread in inoculated leaves. However, PPV was able to move systematically in transgenic tobacco overexpressing SA-degrading enzymes [58]. In *Arabidopsis*, SA accumulates in *cpr1* and *cpr5* mutants due to the absence of negative regulators of the SA metabolic pathway, and the long-distance movement of CaMV is blocked in these mutants [59]. In wheat, *TaLIP* positively regulates the resistance of wheat to WYMV [54]. The promoter sequence analysis showed that there were nine copies of the ABA-responsive elements in the promoter, and the expression level of *TaLIP* was induced by exogenous SA [54]. The transcription level of the ABA signaling pathway genes *TaABI5*, *TaABI8*, *TaPYL1*, and *TaPYL3* were suppressed in *TaLIP*-silenced wheat [54]. In this study, the IAA, GA, SA and ABA signaling pathway genes were induced or suppressed in *DvGELP53*-silenced *D. villosum*, indicating that *DvGELP53* may regulate the long-distance movement of viruses through hormone signals. Taken together, our work revealed that *DvGELP53* inhibits long-distance BSMV movement, possibly through the hormone signals pathway. The mechanism of DvGELP53 participation in the virus movement requires further research.

## 4. Materials and Methods

### 4.1. Plant Materials

*D. villosum* (genome VV, accession No. 91C43) from the Cambridge Botanical Garden, Cambridge, UK, was used for gene cloning and expression analysis. Powdery mildew susceptible variety Sumai 3 was used for propagation of fresh spores of powdery mildew isolate E26. *N. benthamiana* (NC89) plants were used for subcellular localization analysis. The materials were grown in a greenhouse, and the growth conditions were as follows: 14/10 h day/night cycle, 24/20 °C day/night temperature, 8000 lux light intensity and 70% relative humidity.

### 4.2. Identification of the DvGELP Genes in D. villosum

The identification of the *DvGELP* gene family in *D. villosum* was performed according to Zhang et al. (2020) with minor modifications [60]. The GDSL lipase protein in the annotated protein database of *D. villosum* (data not shown) was searched and entries containing gene ID and protein sequences were obtained. A conserved domains (CD) search (https://www.ncbi.nlm.nih.gov/Structure/cdd/wrpsb.cgi (accessed on 15 February 2021)) was performed to identify the protein sequences that contain the GDSL-conserved domain (PF00657). After validation, all the candidate genes encoding the GDSL domain were identified in the *D. villosum* genome.

### 4.3. Phylogenetic Analysis of the DvGELP Gene Family

Phylogenetic analysis was performed as described by Lai et al. (2017) with the following modifications [4]: multiple sequence alignment was conducted using ClustalW, which was integrated in MEGA X; the neighbor-joining analysis was performed in MEGA X using a p-distance parameter with 1000 bootstrap replications.

Chromosomal location of the predicted *DvGELP* genes from *D. villosum* was obtained by blasting to the genomic sequence using the cDNA sequences as a query. Then, their locations were drawn onto the physical map of each chromosome by using Tbtools [61].

The gene structure and intron insertion sites of the *DvGELP* genes were obtained from the *D. villosum* database (data not shown), and the domains of the DvGELPs were predicted by CD search (https://www.ncbi.nlm.nih.gov/Structure/cdd/wrpsb.cgi (accessed on 24 February 2021)). The corresponding evolutionary trees were constructed using MEGA X as described above. The formation of the protein structure was constructed using Tbtools [61].

### 4.4. Subcellular Localization

The subcellular localization of DvGELP53 was performed according to Zhang et al. (2020) with some modifications [60]. The CDS of DvGELP53 were cloned into vector pCambia1305-GFP to produce the fusion expression construct 1305-GFP:DvGELP53. The plasma membrane marker construct (mCherry-SYP122) was provided by Prof. Yiqun Bao. The 1305-GFP:DvGELP53 fusion construct and mCherry-SYP122 were transformed into *N. benthamiana* epidermal cells by *Agrobacterium tumefaciens* (strain GV3101) incubated in darkness at 22 °C for 48–60 h. The fluorescence signals were observed under confocal microscopy (LSM780, Zeiss, Oberkochen, Germany).

### 4.5. Transcription Abundance Analysis

Plant treatments were performed as described by Zhao et al. (2018) and Zhang et al. (2020b) with modifications [60,62]. The seedlings of *D. villosum* were grown in a liquid or soil until the three-leaf stage. For testing the transcript levels of *DvGELP53* in different tissues of *D. villosum*, the roots, stems, leaves and immature spikes were sampled at 20 d after flowering. For the heat, cold and drought treatment, the plants were transferred to 42 °C and 4 °C or dipped into 20% PEG 6000, and the leaves were sampled at 0, 1, 2, 4, 8, 12, 24 and 48 h after treatment. For powdery mildew treatment, the plant was inoculated with pathogen isolate E26, and the leaf tissues were sampled at 0, 2, 6, 12, 24, 36, 48 and 72 h after inoculation. For chitin, flg22, phytohormones and H_2_O_2_ treatments, the plants were sprayed with 100 μg/mL insoluble chitin, 100 μg/mL flg22, 5 Mm SA, 0.2 mM ABA, 7 mM H_2_O_2_ and 0.1 mM ET, respectively, and all the leaf tissues were collected at 0, 1, 2, 4, 8, 12, 24 and 48 h after spraying. For the NaCl and Cd treatments, the plants were transferred to 1/2 Murashige and Skoog (MS) medium with 1 mM CdCl_2_ or 100 mM NaCl and all the leaf tissues were collected at 0, 1, 2, 4, 8, 12, 24 and 48 h after treatment. All of the samples were rapidly frozen in liquid nitrogen, then stored in an ultra-freezer (−80 °C) until use.

Total RNA was extracted using the TRIzol reagent according to the manufacturer’s protocol (Invitrogen). Total RNA (2 µg) per sample was used to synthesize the first-strand cDNA using an RNA PCR Kit (TaKaRa). Specific primers for *DvGELP53* were used for qRT-PCR (Table S2). The tubulin gene was used as the internal control for normalization; qRT-PCR was performed with AceQ SYBR Green (Vazyme) using a Louts PCR 480 sequence detection system. The relative value of gene expression was derived by 2^–^^△△CT^ [63].

### 4.6. Virus-Induced Gene Silencing (VIGS)

A partial DvGELP53 sequence was amplified by primer pairs (Table S2), and the resulting fragment (174 bp) was reversely inserted into the vector BSMV:γ to generate the recombinant vector BSMV:DvGELP53.

VIGS was performed as described by Wang et al. (2010) [64] with modifications. Recombinant RNAγ cDNA clones were in vitro transcribed to obtain the RNAγ and RNAγ:DvGELP53 transcripts. The methods for in vitro transcription and BSMV recombinant virus inoculation were described by Zhou et al. (2007) [65]. After inoculation, photobleaching on plants was checked at regular intervals. The normal image and the photochemical utilization (Y(II)) of BSMV:γ and BSMV:DvGELP53 image were detected at 10, 20 and 30 days after inoculation using a camera and Dual-PAM-100 according to Escher et al. (2006) [66].

### 4.7. Transcriptome Analysis

The *D. villosum* plants were grown in field and the root, leaf, node, bud, stem, spike and grain were sampled at 20 d after flowering. The virus with BSMV:DVGELP53-inoculated *D. villosum* was collected at 7 days after virus inoculation, and the virus with BSMV:γ-inoculated *D. villosum* was used as mock control. Each sample had three biological replicates. All of the samples were immediately frozen in liquid nitrogen and stored at −80 °C. Total RNA was extracted using the TRIzol reagent (Invitrogen, Waltham, MA, USA) according to the manufacturer’s instructions. RNA concentration was determined using a NanoDrop spectrophotometer and 1.2% agarose gel electrophoresis. RNA-Seq was performed using a BGISEQ-500 sequencing platform by BGI Company, Shenzhen, China. The raw reads were cleaned by removing the adapter and low-quality sequences (percentage of low-quality bases with a quality value ≤ 10 in more than 20% of a read). The gene expression levels were calculated by RSEM (v1.2.8, default). The DEGs (differentially expressed genes) were detected by DEGseq (fold change ≥ 2 and adjusted *p-*value ≤ 0.001). The GO enrichment analysis and KEGG enrichment analysis of DEGs were performed using a free online platform for data analysis, the OmicShare tools (http://www.omicshare.com/tools (accessed on 5 April 2021)). Log2 FPKM values after Z-score normalization were used for the heatmap of DEGs expression.

## 5. Conclusions

This study identified 193 *GELP* genes from *D. villosum*. Evolutionary and protein structure analyses showed that *DvGELP* could be classified into 11 groups, and closer *DvGELP* genes in the phylogenetic tree have similar gene structures. The *cis*-elements in the primer sequence and tissue-specific expression analysis showed that *GELP* genes may be involved in multiple pathways of plant development. *DvGELP53* was upregulated in the leaves of *D. villosum* in response to multiple biotic and abiotic stresses. Transient silencing of *DvGELP53* inhibits long-distance BSMV movement. Our study systematically analyzed the distribution and evolution of the *GELP* gene family in *D. villosum* and demonstrated the important role of *DvGELP53* in plant–virus interaction.

## Data Availability

Not applicable.

## Supplementary Materials

The following are available online at https://www.mdpi.com/article/10.3390/ijms222212317/s1.
