# Supplementary material for: Genome-Wide Identification of GDSL-Type Esterase/Lipase Gene Family in Dasypyrum villosum L. Reveals That DvGELP53 Is Related to BSMV Infection
Source: Int J Mol Sci. 2021 Nov 15;22(22):12317. doi: 10.3390/ijms222212317 (PMC8624868; doi:10.3390/ijms222212317)
Supplement: Supplementary file 1 [file ijms-22-12317-s001.zip › ijms-1457156-supplementary/ijms-1457156-supplementary.pdf]

International Journal of
Molecular Sciences

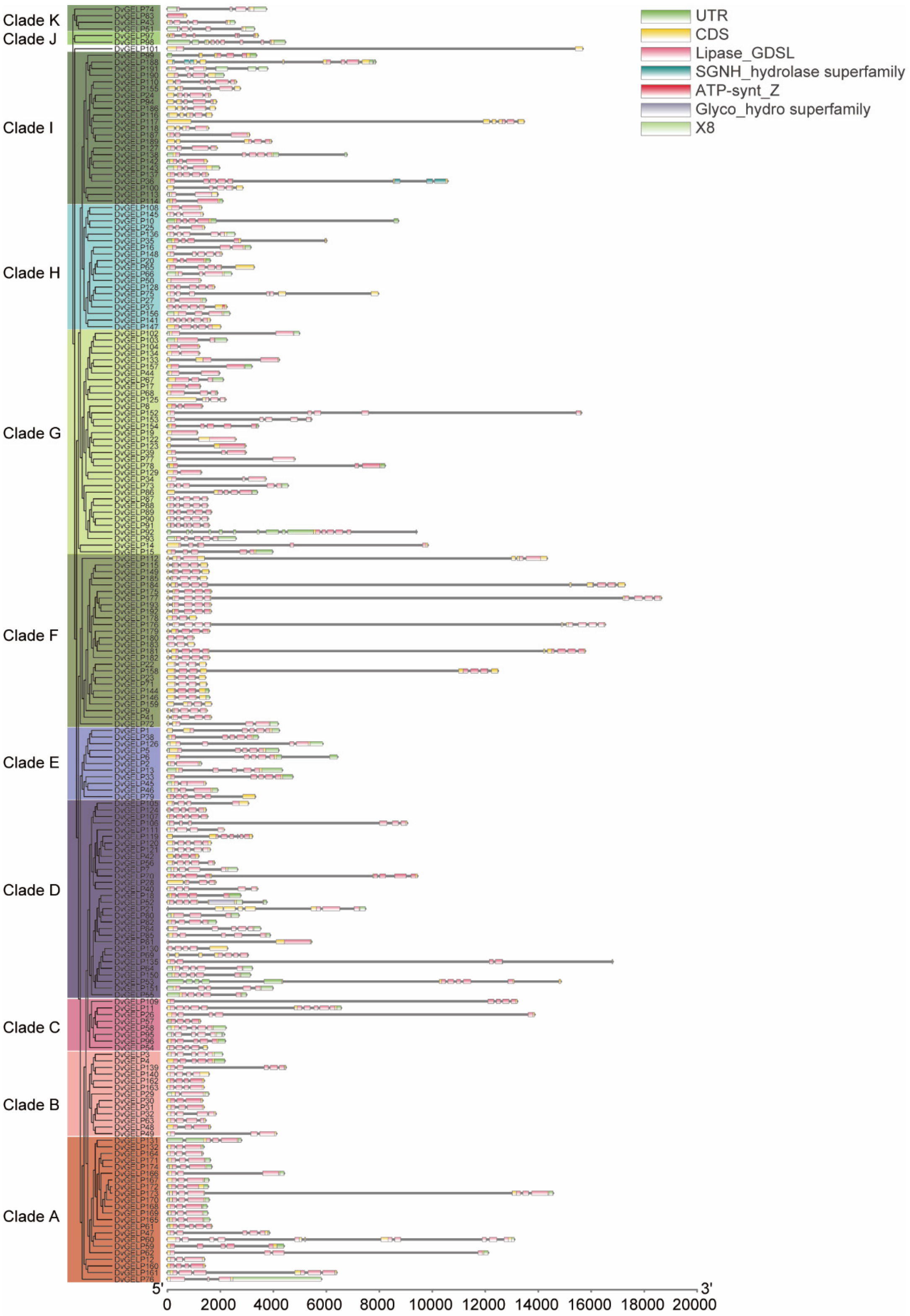

Figure S1. Phylogenetic analysis and protein structures of the *DvGELP* gene family in *D. villosum*. The left and the right represent the phylogenetic tree and gene structure, respectively. Multiple protein domains, exon, and intron were represented by colored boxes. Legends are shown.

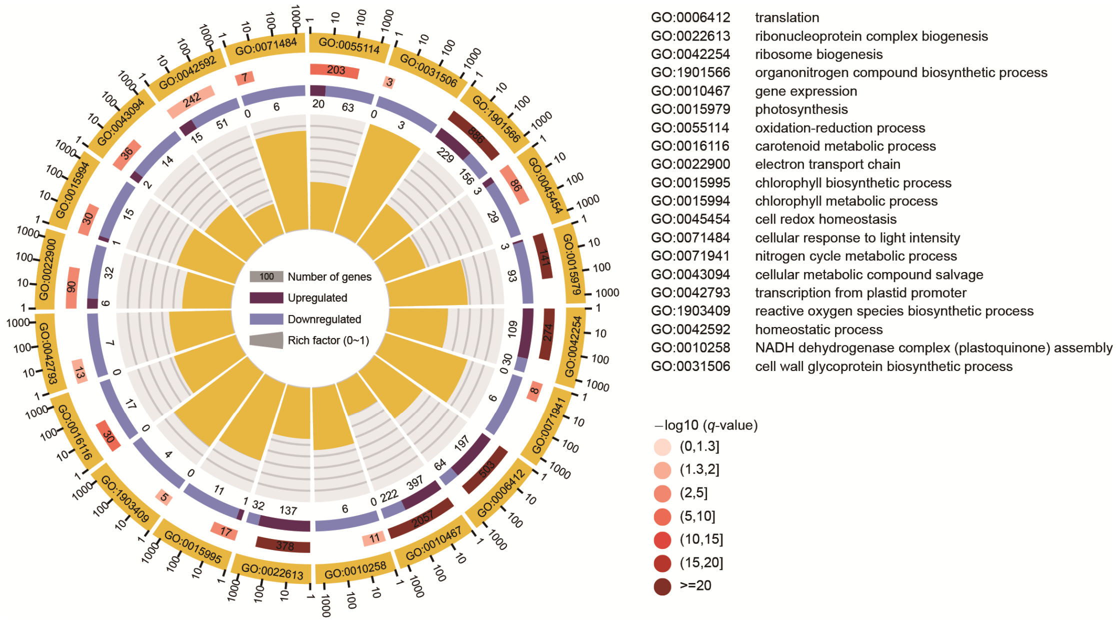

Figure S2: GO enrichment analysis of BSMV:γ and BSMV:DvGELP53

GO enrichment of DEGs of BSMV:γ and BSMV:DvGELP53 for the third leaves inoculated with virus. The circular chart represents distinct GO term of DEGs. The graph is divided into four circles from the outside to the inside. The first circle represents the terms of enrichment and the outside of the circle is the coordinate ruler of the number of genes. The second circle represents the number of background genes in the category and the *q*-value. The length of the bar represents the number of genes and the shade of red represents the size of the *q*-value. The third circle represents the ratio of the number of upregulated and downregulated genes, dark purple and light purple represent upregulated and downregulated genes, respectively, and the number of genes is shown below. The fourth circle represents the rich factor value of each category, and each box on the background represents 0.1.
